# Extended bioreactor conditioning of mononuclear cell–seeded heart valve scaffolds

**DOI:** 10.1177/2041731418767216

**Published:** 2018-04-10

**Authors:** Mitchell VeDepo, Eric Buse, Rachael Quinn, Richard Hopkins, Gabriel Converse

**Affiliations:** 1 Cardiac Regenerative Surgery Research Laboratories of The Ward Family Heart Center, Children’s Mercy Kansas City, Kansas City, MO, USA; 2 Bioengineering Program, The University of Kansas, Lawrence, KS, USA; 3 Division of Cardiac Surgery, University of Maryland School of Medicine, Baltimore, MD, USA

**Keywords:** Tissue-engineered heart valve, mononuclear cells, bioreactor conditioning

## Abstract

The tissue-engineered heart valve may be the ideal valve replacement option but still must overcome challenges in leaflet recellularization. This study sought to investigate the potential for leaflet matrix restoration and repopulation following mononuclear cell seeding and extended periods of bioreactor conditioning. Human aortic heart valves were seeded with mononuclear cells and conditioned in a pulsatile bioreactor for 3 days, 3 weeks, or 6 weeks. The results of this study determined that a mononuclear cell population can be readily localized within the leaflet tissue in as little as 3 days. Furthermore, as extended bioreactor condition continued to the 3- and 6-week time points, the mesenchymal stem cell subfraction proliferated and appeared to become the predominant cell phenotype. This was evident through positive expression of mesenchymal stem cell markers and no expression of mononuclear cell markers observed by immunohistochemistry in the 3- and 6-week groups. In addition, cells in the 3- and 6-week groups exhibited an up-regulation of mesenchymal stem cell–associated genes (*THY1, NT5E*, and *ITGB1*) and a down-regulation of mononuclear cell–associated genes (*CD14, ICAM1*, and *PECAM1*) compared to the initial seeded cell population. However, repopulation of the leaflet interstitium was less extensive than anticipated. Valves in the 6-week time point also exhibited retracted leaflets. Thus, while the 3-week bioreactor-conditioning period used in this study may hold some promise, a bioreactor-conditioning period of 6 weeks is not a viable option for clinical translation due to the negative impact on valve performance.

## Introduction

Heart valve disease is a common cause of global morbidity with etiologies ranging from congenital to degenerative.^1^ Although a variety of options are available for heart valve replacement, including mechanical valves, bioprosthetic xenografts, and cryopreserved homografts, the ideal heart valve substitute has yet to be developed.^2^ The tissue-engineered heart valve (TEHV) offers great potential as a permanent valve substitute for disorders requiring valve replacement, overcoming the limitations of the other replacement options. This is particularly true for the pediatric population for which an initial intervention using a living, growing valve would eliminate the need for multiple revision surgeries and catheter interventions as the child matures.^2^ However, technical barriers and regulatory challenges have hindered the development of a clinically useful TEHV, such as creating a functional valve scaffold and the repopulation of the valve scaffold with an autologous cell population. Decellularized heart valves are a promising option for a functional valve scaffold, as they retain the structure and mechanical anisotropy of the native valves from which they are derived.^3–5^ However, implantation of decellularized heart valves in clinical use and in ovine models has demonstrated only partial recellularization of the valve, with the distal to mid regions of the interstitial tissue of the valve leaflet remaining notably acellular.^6–8^ The repopulation of the valve scaffold remains a critical challenge, since an autologous cell population is required to provide growth and remodeling of the tissue-engineered construct, thereby preventing further revision surgeries. The careful selection of an appropriate cell line for seeding heart valve scaffolds may overcome the technical barrier of decellularized scaffold repopulation by differentiation into an appropriate cell type and/or through the release of trophic factors to increase host-cell repopulation. The ideal cell source should (1) be patient specific and readily available in the clinical setting, (2) require minimal manipulation prior to scaffold seeding, and (3) offer the potential for phenotypically appropriate scaffold repopulation, either through direct proliferation of the seeded cells (prior to or following implantation) or through chemotactic recruitment of autologous cells (following implantation).^9–11^

Traditional approaches to heart valve tissue engineering have generally employed extended periods of cell culture and valve conditioning in custom bioreactors with the aim of creating end-stage living tissue ex vivo before theoretical or animal implantation. These approaches have used numerous cell types and sources including endothelial cells and myofibroblasts, often isolated from arterial, venous, or leaflet tissue.^10,12–14^ Despite varying degrees of success in the research arena, clinical application of these cell sources is challenged by donor site morbidity from cell harvest. Bone marrow mesenchymal stem cells (MSCs) are an alternative option for valve seeding, providing autologous cells with pluripotent differentiation capabilities.^15^ However, considering the low proportion of MSCs within bone marrow (0.002%–0.02%), lengthy periods of in vitro cell expansion are required prior to valve seeding to ensure adequate cell coverage.^16^

Bone marrow–derived mononuclear cells (MNCs) are beginning to receive interest as a candidate cell population for scaffold seeding. A group of European researchers have reported on the seeding of polymeric heart valve scaffolds with MNCs using fibrin as a cell carrier.^17–19^ This strategy relies on the migration of seeded cells from the fibrin carrier into the polymer scaffold after implantation, followed by further recruitment of autologous cells through a paracrine signaling mechanism. Four weeks after implantation of these scaffolds in primates, seeded MNCs were no longer present; however, the presence of autologous endothelial and interstitial cells was observed.^17–19^ Work by Roh et al.^20^ provides further evidence that a “pilot” population of MNCs may act to recruit autologous cells, as the authors reported that MNCs seeded on polymeric vascular grafts prior to implantation in mice were no longer detectable after only 1 week in vivo, though overall cellularity was maintained due to the infiltration of autologous monocytes followed by scaffold repopulation with host smooth muscle cells and endothelial cells.

Of particular interest when seeding with MNCs is the persistence of MNC subpopulations, such as MSCs, macrophages, and leukocytes, throughout extended conditioning periods. Since MSCs are commonly isolated from bone marrow through cell culture, it is possible that MNC seeding and extended culture will lead to expansion of the MSC population, effectively skipping traditional cell culture methods. All afore mentioned studies seeding with MNCs have used animal implant models to assess the potential for autologous recellularization, yet the outcome of a seeded MNC population throughout extended ex vivo valve conditioning remains unknown. The fate of a seeded MNC population without the effect of the host inflammatory response is unclear. In addition, preliminary studies within our lab have indicated that bioreactor conditioning may be used to drive cellular infiltration into the valve leaflet, leading to better leaflet recellularization. Therefore, the purpose of this study is to investigate the fate of an MNC population seeded onto decellularized heart valves over an extended period of ex vivo processing. We have previously shown the ability to localize an MSC population into the leaflet matrix of a decellularized valve scaffold using a novel bioreactor and pressure conditioning. Using similar methods, it is expected that the seeded MNCs in this study will localize within the leaflet tissue of the decellularized valves and extended conditioning will lead to proliferation of the MSC population.

## Methods

### Tissues and tissue processing

Human aortic valves were obtained from a tissue-banking facility (LifeNet Health) and stored in a cryopreserved state until use. The cryopreservation procedure is modeled after clinical tissue-handling protocols and freezes the valves at 1°C/min using a controlled rate freezer (2100 Series, Custom Biogenic Systems) before being stored in a cryofreezer at −180°C. Decellularization was performed as described previously.^21^ Briefly, aortic valves were subjected to reciprocating osmotic shock, followed by detergent (Triton X-100, sodium-lauroyl sarcosine) and enzymatic (Benzonase) washes to remove cellular material. Extraction of organic material was performed using recirculating water and ion exchange resins. After decellularization, the valves were again cryopreserved using the above procedure and stored at −180°C until valve seeding.

### Cells and valve seeding

Human bone marrow was obtained from a commercial source (Lonza) and the MNC fraction was isolated using a bone marrow filter system (Kaneka).^22^ The decellularized valves were thawed from cryopreservation and then seeded with the entire mononuclear fraction isolated from the bone marrow using an established protocol.^23^ Briefly, isolated MNCs were suspended in 10 mL of media and seeded into the lumen of the valve which is mounted in a static bioreactor chamber containing valve media (Dulbecco’s Modified Eagel Medium: Nuitrient Mixture F12 (Life Technologies) and 10% fetal bovine serum (FBS)). After introduction of the cells, the seeded valve was incubated in static culture (37°C, 5% CO_2_) for 24 h to allow cell adhesion.

### Valve bioreactor conditioning

After 24 h static culture, the seeded valve was transferred to a pulsatile bioreactor chamber containing valve media, and the assembly was placed in a linear actuator to create cyclic positive and negative pressure profiles within the bioreactor chamber, as described previously.^23^ In all groups, the valves were cultured in a cyclic negative pressure profile (–20 to 5 mmHg) for 2 days, after which the 3-day group (1 day static + 2 days negative; n = 6) of valves were removed from culture, and the valve leaflets were dissected for analysis. The remaining two groups were then switched to cyclic positive pressure profile (–5 to 50 mmHg) and cultured for an additional 18 days (3-week group; n = 6) or 39 days (6-week group; n = 3) before dissection and analysis. The flow rate of culture media within the valve conduit is estimated to be 90 and 310 mL/min for negative and positive pressure profiles, respectively. Upon dissection, samples from the valve leaflets were designated for mechanical testing and biochemical assays or histology, immunohistochemistry (IHC), and real-time polymerase chain reaction (rt-PCR) analysis.

### Histology, IHC, and PCR

Samples for histology were sectioned along the radial plane of the leaflets from the 3-day, 3-week, and 6-week conditioned valves. Hematoxylin and eosin (H&E) staining was used to evaluate repopulation of the decellularized valve leaflets, specifically by the presence of cells within the leaflet tissue and on the leaflet surface. Movat’s pentachrome staining was used to evaluate the biochemical composition of the leaflet extracellular matrix. Protein expression of the seeded cells was evaluated by IHC. Unstained slides were blocked in 10% normal goat serum for 1 h before overnight incubation at 4°C with 1:100 diluted primary antibodies. The primary antibodies (Abcam) targeted alpha smooth muscle actin (αSMA), heat shock protein 47 (HSP47), CD29, CD90, CD45, CD34, CD68, and vimentin (VIM). Fluorescent secondary antibodies (Alexa Fluor 488 or Alexa Fluor 594; Life Technologies) were then incubated for 1 h followed by nuclear counterstaining (DAPI; Life Technologies).

Gene expression was measured by rt-PCR analysis and was evaluated as the relative fold change in gene expression compared to the pre-seeding cell population. Cell samples were taken immediately before valve seeding and tissue samples from each leaflet were taken during valve dissection and frozen in liquid nitrogen. The leaflet tissue from each valve was combined to form one sample (n = 3 per group) to indicate the gene expression across the valve. The frozen tissue was pulverized and the total RNA was isolated using the RNeasy Micro Kit (Qiagen) accompanied by DNase digestion (DNA-free™ kit; Invitrogen). The RNA was then reverse transcribed to complementary DNA (cDNA) using the High-Capacity cDNA Reverse Transcription Kit (Invitrogen). rt-PCR (7300 Real-Time PCR System; Applied Biosystems) was performed using a custom phenotyping array (TaqMan; Life Technologies). Polymerase chain reaction (PCR) data are conveyed as the relative fold change in gene expression between the matched, pre-seeding cell population, and the cell population in the valve after bioreactor conditioning.

### Mechanical testing

The mechanical behavior of the leaflet tissue from the 3-day group and 3-week group of tissue-engineered valves was measured using biaxial loading. The 6-week group of tissue-engineered valves was not included for mechanical testing due to the lack of coaptation between leaflets. Biaxial mechanical testing was performed using methods described previously.^3^ Rectangular specimens (9 mm × 6 mm, n = 9) were cut from the belly region of each leaflet and mounted on a four motor equibiaxial loading system submerged in phosphate-buffered saline at 37°C (LM1 TestBench, Bose ElectroForce). Samples were mounted so that the loading axes were aligned with the radial and circumferential directions of the sample. All samples were then loaded to an equibiaxial membrane tension of 60 N/m using a rise time of 10 s. The directional peak stretch ratios were calculated from strain data in the circumferential $\left(\lambda_{C}^{\mathrm{peak}}\right)$ and radial $\left(\lambda_{R}^{\mathrm{peak}}\right)$ directions. Areal strain was used as a measure of net extensibility and was calculated from equibiaxial data as $(\lambda_{C}^{\mathrm{peak}}\times \lambda_{R}^{\mathrm{peak}}-1)\times 100\%$. Results were compared against previously reported biaxial mechanical data from cryopreserved and decellularized valves.^4^

### Biochemical

The collagen and sulfated glycosaminoglycan (GAG) concentrations of the tissue-engineered leaflet extracellular matrix from the 3-week group and 6-week group was measured using colorimetric assays. The QuickZyme Total Collagen Assay (QuickZyme Biosciences) was used to measure the collagen concentration. Samples (approx. 15 mg, n = 9) were prepared by overnight hydrolysis in 6 M HCl at 90°C then prepared following the manufacturers protocol. The hydroxyproline concentration was measured using an ultraviolet (UV)–visible spectrophotometer (VersaMax, Molecular Devices) at 570 nm. The GAG concentration was measured using the Blyscan Sulfated Glycosaminoglycan Assay (Biocolor). Tissue samples (approx. 15 mg, n = 9) were processed according to manufacturer’s protocol, and the results were measured using a UV–visible spectrophotometer at 656 nm. The collagen and GAG concentrations are reported as µg/mg of wet tissue.

### Statistical analysis

Statistical analysis of normally distributed data was performed using one-way analysis of variances (ANOVA; SigmaStat 3.5, Systat Software, Inc.), and post hoc comparisons between groups were performed using the Holm–Sidak test. Differences were considered statistically significant at p < 0.05. Error values are reported as the standard deviation of the mean.

## Results

### Tissue processing and valve seeding

As observed in previous studies, decellularization resulted in complete removal of cells from the valve tissue while preserving the overall extracellular matrix architecture (data not shown).^4^ Following MNC seeding and bioreactor conditioning, the gross appearance of the aortic valves in the 3-day group and 3-week group was normal with functional leaflets capable of coaptation. Valves in the 6-week group were incompetent due to retracted leaflets, though otherwise appeared normal.

### Histology

**Figure 1.**
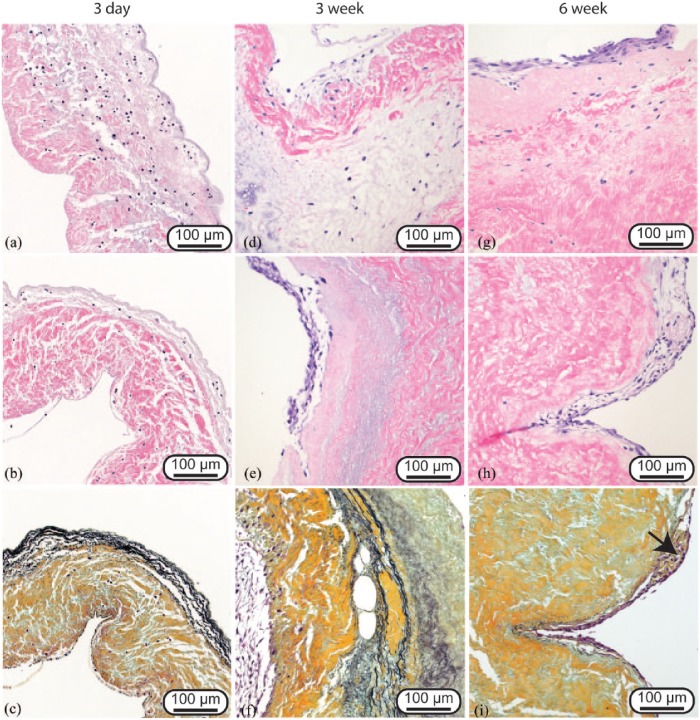
Representative H&E (a, b, d, e, g, and h) and Movat’s pentachrome (c, f, and i) images of 3-day (a–c), 3-week (d–f), and 6-week (g–i) bioreactor-conditioned heart valve leaflets. Three-day-conditioned leaflets exhibited high interstitial recellularization. Three-week- and 6-week-conditioned valves exhibited decreased interstitial recellularization but increased numbers of cells on the leaflet surface, often in multilayered clumps. Movat’s pentachrome staining demonstrated preservation of the leaflet structure (collagen, gold; elastin, black; and GAG, blue). There was evidence of collagen deposition within some samples of the 6-week-conditioned valves (black arrow).

Extended conditioning in the bioreactor of the MNC-seeded heart valves led to differences in the recellularization of the valve leaflets, as seen by histology (Figure 1). Three-day processing (1 day static and 2 days negative pressure) led to localization of cells within the interior of the leaflet tissue, though no cells were present on the surface of the leaflet (Figure 1(a) and (b)). After 3 weeks of processing (1 day static, 2 days negative pressure, 18 days positive pressure), cells were still present within the leaflet tissue as well as on the surface of the leaflet (Figure 1(d) and (e)). The cells on the surface of the 3-week group were often multilayered and clumped together. After 6 weeks of bioreactor conditioning (1 day static, 2 days negative pressure, 39 days positive pressure), there was a decrease in the number of cells within the leaflet tissue, though more cells were present on the leaflet surface (Figure 1(g) and (h)). Similar to the 3-week group, the cells on the surface of the leaflets in the 6-week group were multilayered and clumped together. Movat’s pentachrome staining revealed the preservation of collagen, GAG, and elastin within the extracellular matrix for all bioreactor-conditioning time points (Figure 1(c), (f) and (i)). In addition, there was evidence of collagen production by the seeded cells in the 6-week group (Figure 1(i)).

### Protein and gene expression

**Figure 2.**
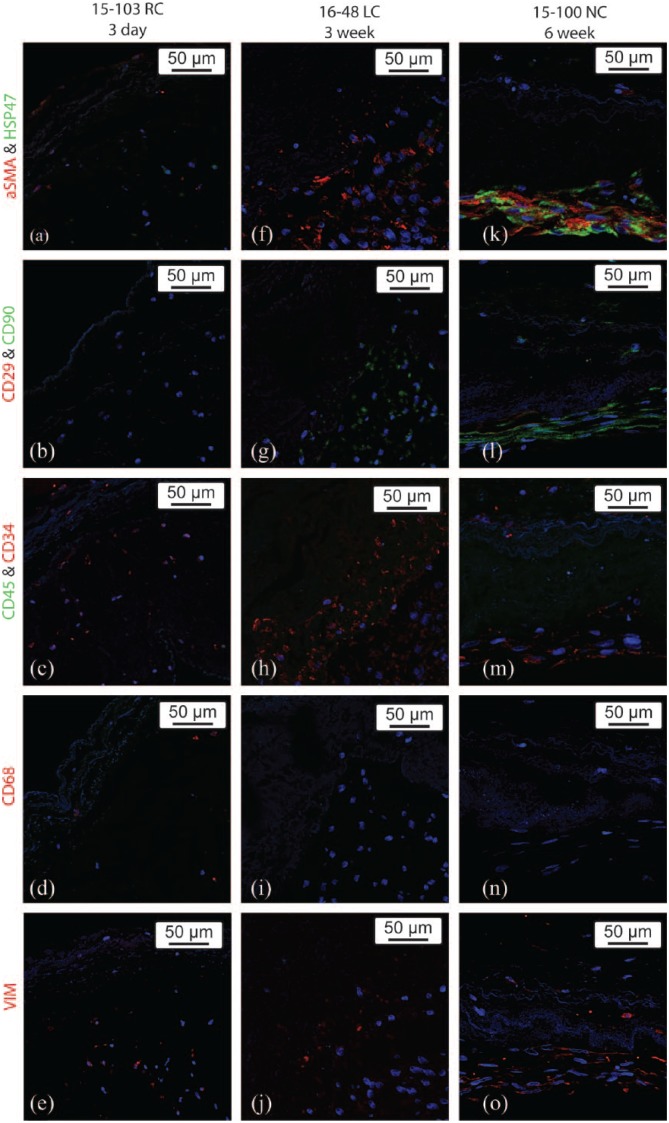
Representative IHC-stained sections of 3-day (a–e), 3-week (f–j), and 6-week (k–o) bioreactor-conditioned heart valve leaflets. Sections were stained for αSMA (red) and HSP47 (green; a, f, and k), CD29 (red) and CD90 (green; b, g, and l), CD45 (green) and CD34 (red; c, h, and m), CD 68 (red; d, i, and n), and VIM (red; e, j, and o).

IHC revealed a changing cell phenotype with extended periods of bioreactor conditioning (Figure 2). Previous work has shown that an MNC population isolated from bone marrow initially consists of macrophages, leukocytes, hematopoietic stem cells, and a small population of MSCs.^16^ In this study, the cells on the 3-day group of seeded valves had protein expression that closely resembled the profile expected from isolated MNCs. Specifically, cells seeded on the 3-day group had positive expression of CD34 and CD68, partial expression of αSMA, HSP47, and VIM and notably were negative for CD29 and CD90 (Figure 2(a)–(e)). As the duration of bioreactor conditioning increased to 3 weeks, the protein expression began to more closely resemble an MSC population (Figure 2(f)–(j)). Compared to the 3-day group, the 3-week group had increased expression of αSMA, CD90, and CD34 with decreased expression of CD68. The protein expression in the 6-week group further resembled an MSC population with a high expression of HSP47, greater expression of αSMA, CD90, and VIM, and decreased expression of CD68 and CD34 (Figure 2(k)–(o)).

**Figure 3.**
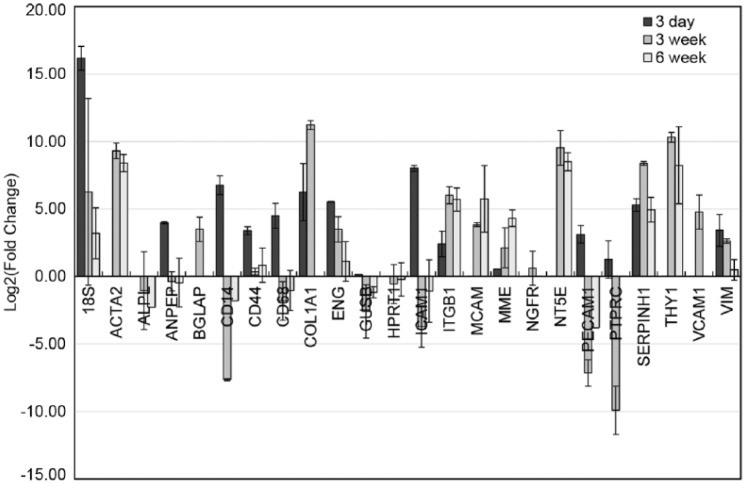
Relative fold change in the gene expression between 3-day-, 3-week-, and 6-week-conditioned heart valves in relation to their respective pre-seeded cell populations. Changes in gene expression are displayed as the Log2 of the average relative fold change within a group. Error bars represent the standard deviation.

Gene expression by rt-PCR analysis agreed with the changing phenotype observed by IHC (Figure 3). The cells in the 3-day group showed up-regulation of immune-responsive cells compared to the initial seeding population (ANPEP, CD14, CD44, CD68, ICAM1, and PTPRC) and little to no up-regulation of MSC or valve interstitial cell (VIC) related markers. However, with increased bioreactor conditioning, the valves in the 3-week and 6-week groups had generally increased regulation of the MSC- or VIC-related markers compared to the seeding population (ACTA2, ITGB1, MCAM, NT5E, SERPINH1, and THY1). The endothelial cell marker (PECAM1) exhibited a slight up-regulation in the 3-day group, but showed markedly decreased regulation in the 3-week and 6-week groups, suggesting there was no significant endothelial cell differentiation. Additional gene markers were not displayed in Figure 3 because they had no expression in either the cells or the tissue. These include ADIPOQ, CD19, CD34, and tumor necrosis factor (TNF) which were expressed in the seeded cell populations but was not expressed by any of the valve groups. In addition, ITGA11 and MMP1 were expressed by the 3-week valves but had no expression in the corresponding cell populations at the time of seeding.

### Mechanical testing

**Figure 4.**
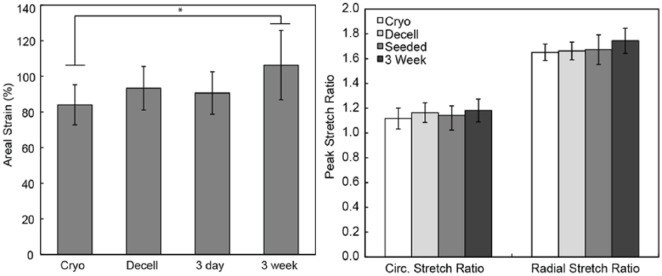
Mechanical properties, areal strain, and directional peak stretch ratios of the 3-day- and 3-week-conditioned heart valve leaflets. Previously reported data for cryopreserved (cryo) and decellularized (decell) human aortic valves are included for comparison [ref]. *Statistically significant difference (p < 0.05).

Mechanics of the TEHV leaflets from the 3-day and 3-week groups were compared against each other and against leaflet samples from cryopreserved and decellularized human aortic valves (Figure 4).^4^ Leaflets from the 6-week group of tissue-engineered valves were not included for mechanical testing due to incompetence in the leaflets after bioreactor culture. The areal strain of the valve leaflets from the 3-day group was 90.63% ± 11.90%, and the peak stretch ratios were 1.14 ± 0.08 and 1.67 ± 0.12 in the circumferential and radial directions, respectively. Valve leaflets from the 3-week group of tissue-engineered valves had an areal strain of 106.30% ± 19.46% and peak stretch ratios of 1.18 ± 0.09 and 1.74 ± 0.10 in the circumferential and radial directions, respectively. The only significant difference between groups in the measured areal strain was between the cryopreserved and 3-week group (p = 0.012). All other groups were statistically similar (p > 0.05), and no significant differences were measured between groups in the directional peak stretch ratios (p > 0.05).

### Biochemical analysis

Biochemical assays measured the collagen and GAG concentration from valves in the 3-week and 6-week groups, and those results were compared against previous results from cryopreserved and decellularized valves.^4^ The 3-day group was not expected to exhibit matrix remodeling in such a short time and was therefore excluded. Cell seeding with MNCs and extended bioreactor conditioning did not have a significant effect on the biochemical composition of the decellularized heart valve leaflets (Table 1). The collagen concentration between all groups was found to be statistically similar (p > 0.05). As previously reported, decellularization causes a loss of GAGs within the leaflet tissue and a significant decrease in GAG concentration is found between decellularized and cryopreserved samples. Seeding with MNCs and extended bioreactor conditioning did not have a significant effect on the GAG concentration, since no significant difference was found between decellularized and tissue-engineered valves (p > 0.05). In addition, the cryopreserved valves retained a significantly greater GAG concentration than the tissue-engineered valves from the 3-week group (p < 0.001) and 6-week group (p < 0.001).

**Table 1.** Biochemical data for 3 week and 6 week conditioned heart valve leaflets. Previously reported data for cryopreserved (cryo) and decellularized (decell) human aortic valves are included for comparison [ref]. Data reported as the mean ± standard deviation.

Test group	Collagen (µg/mg wet tissue)	GAG (µg/mg wet tissue)
Cryo	48.24 ± 4.92	1.73 ± 0.41*
Decell	44.33 ± 11.59	0.78 ± 0.21*
3-week	47.89 ± 14.82	0.58 ± 0.25
6-week	39.73 ± 11.22	0.64 ± 0.23

GAG: glycosaminoglycan.

* indicates a significant difference between groups (*p* < 0.05).

## Discussion

The purpose of this study was to investigate the fate of MNCs isolated from bone marrow seeded onto decellularized heart valve scaffolds. Specifically, we sought to evaluate the persistence of subpopulations such as MSCs, macrophages, and leukocytes on and within the valve leaflet following extended periods of bioreactor conditioning. Since MSCs are commonly isolated from bone marrow through cell culture, it was expected that MNC seeding and extended conditioning will lead to expansion of the MSC population, effectively skipping traditional cell culture methods.

The results of this study yielded both expected and unexpected outcomes. As expected, MNCs can be quickly localized into the leaflet matrix. There is evidence of MNC seeding leading to rapid recellularization in situ, and we now have evidence that such rapid recellularization can occur in vitro.^17^ It was also expected that extended bioreactor processing would lead to proliferation of the MSC subpopulation from the seeded MNCs. This was demonstrated by the increased expression of MSC-associated genes following extended periods of bioreactor conditioning in both the 3-week and 6-week processing groups. Conversely, macrophage markers were generally down-regulated with increased processing times. This effect was also observed by IHC staining, which identified CD34-, CD45-, and CD68-positive cells in the 3-day group, indicating a mixed-cell population of hematopoietic stem cells, leukocytes, and macrophages. On the other hand, cells in the 3-week and 6-week groups showed positive expression of CD90, CD29, αSMA, and HSP47, indicating a population of MSCs and/or myofibroblasts. The positive expression of CD34 during extended bioreactor conditioning may also indicate proliferation of the hematopoietic stem cell population, although there is evidence that MSC population may also express CD34.^24^

Despite proliferation of the MSC population, recellularization of the leaflet matrix following extended bioreactor conditioning was not as extensive as expected. Areas of interstitial repopulation were observed at both the 3-week and 6-week time points; however, a cellularity similar to that of the cryopreserved leaflet was not achieved, and areas devoid of cells were observed. The majority of the cells present on the seeded valves following 3 and 6 weeks of bioreactor conditioning were located on the surface of the leaflet. While MSC proliferation occurred during extended bioreactor processing, extensive localization of MSCs within the leaflet interstitium did not occur. In addition, valve incompetency due to leaflet retraction was observed for these valves. Therefore, the use of long (i.e. 6 weeks) periods of bioreactor conditioning following MNC seeding is likely not a clinically viable heart valve tissue engineering strategy.

MNC seeding and bioreactor processing did not seem to have a significant effect on the mechanics or biochemical properties of the seeded heart valves. The directional strain ratios of all groups remained similar and the 3-week processing group actually displayed an increase in areal strain. Because the mechanism of valves processed for 6 weeks were clearly compromised, we did not perform biaxial mechanical testing for this group, as three additional human aortic valves would have been required and no information regarding the clinical feasibility would have been gained. Biochemical analysis did not reveal any significant changes between decellularized controls and MNC-seeded samples; however, it is worth noting that some collagen deposition was observed within areas of cell localization on surface of the leaflet fibrosa at the 6-week time point (Figure 2(i)). The absence of any significant mechanical or biochemical changes may be due, in part, to the lack of full repopulation of the leaflet interstitium during valve seeding.

As shown by others, MNC-seeded scaffolds have the potential for recellularization in situ. Emmert et al.^17^ and Weber et al.^19^ demonstrated that polymeric heart valve scaffolds seeded with MNCs are capable of recellularization by autologous cells in ovine and primate models, respectively. Roh et al.^20^ proposed that this occurs through an inflammation-mediated mechanism, wherein seeded monocytes attract a population of autologous macrophages which incite a tissue-healing response. The use of MNC seeding onto decellularized scaffolds was explored by Vincentelli et al.^25^ who injected MNCs or MSCs into the base of the leaflet of decellularized pulmonary valves and then implanted into the pulmonary position of juvenile sheep. Seven days after implantation, they observed both groups had seeded cells and recruited host cells present within the scaffold, but at the final explant, the MNC-seeded valves exhibited thickened leaflets, calcification, and CD68^+^ macrophage host cells in the arterial wall.^25^ The results from Vincentelli et al.^25^ favored the MSC group, yet the presence of macrophage cells may align with the inflammation-mediated recellularization mechanism proposed by Roh et al.^20^ However, the calcification and thickened leaflets from their MNC group is troubling. The MSC-favored results by Vincentelli et al.^25^ are further supported by the evidence that seeded MSCs release a cocktail of immunomodulatory proteins that may facilitate in situ recellularization.^26^ Therefore, an MSC pilot population, such as demonstrated in our 3 week bioreactor-conditioning group, may be better suited for valve recellularization compared to a fresh MNC population.

The results from this study provide implications toward the translation of the TEHV, as the mechanism of recellularization, whether in vitro cell proliferation or in situ autologous cell recruitment, determines the extent to which the cell-seeded decellularized heart valve must be processed prior to implantation. For example, matrix repopulation through an inflammatory tissue–healing response, in which the seeded cells serve only to attract autologous cells through paracrine signaling, would mechanistically facilitate brief ex vivo processing protocols. Here, we demonstrated the ability to rapidly localize an MNC population into a decellularized leaflet matrix within 3 days. On the other hand, recellularization through proliferation and differentiation of the seeded population would likely require extended bioreactor processing, though different processing methods should be considered to avoid the observed cell layering on the leaflet surface. The work proposed here segues to future large animal studies (e.g. ovine and papio) to evaluate not only matrix repopulation but also the hemodynamic performance and growth potential of intact TEHVs processed by these methods.

While this study focused on extended conditioning of MNC-seeded heart valve scaffolds in a bioreactor, this creates an inherent limitation such that the in vitro nature eliminates any tissue-healing response that may be expected in vivo. Therefore, a number of factors could not be included in this study that could ultimately affect the recellularization of the valve leaflets, including paracrine and endocrine signaling, the presence of local tissue repair cells, and possible inflammatory responses. The in vivo response fell outside the scope of this study, yet this investigation still provides valuable insight into the persistence of subpopulations during extended conditioning following MNC heart valve seeding.

## Conclusion

This study sought to investigate the potential for leaflet matrix restoration and repopulation following MNC seeding and extended periods of bioreactor conditioning. We found that an MNC population can be readily localized within the leaflet tissue in as little as 3 days. Furthermore, the MSC subfraction within the larger MNC population becomes amplified with prolonged bioreactor conditioning. The effect of MSC proliferation may eliminate the need for the long-term pre-seeding culture of MSC populations, although that time is replaced with extended bioreactor culture. Repopulation of the leaflet interstitium was less extensive than anticipated as extended bioreactor conditioning did not lead to increased interstitial repopulation through cell proliferation; however, this alone does not preclude this TEHV-processing strategy from clinical use, as an incomplete cell population established ex vivo may continue to proliferate after implantation or may provide paracrine signaling to promote further autologous recellularization in vivo. Thus, while the intermediate bioreactor-conditioning period used in this study (i.e. 3 weeks) may hold some promise, a bioreactor-conditioning period of 6 weeks is not a viable option for clinical translation due to the negative impact on valve performance.
